# Comparing the Different Manifestations of Postpartum Mental Disorders by Origin, among Immigrants and Native-Born in Israel According to Different Mental Scales

**DOI:** 10.3390/ijerph182111513

**Published:** 2021-11-02

**Authors:** Shakked Lubotzky-Gete, Maru Gete, Roni Levy, Yaffa Kurzweil, Ronit Calderon-Margalit

**Affiliations:** 1 Hadassah Braun School of Public Health, Hebrew University, Jerusalem 9112102, Israel; ronitcm@gmail.com; 2 Otolaryngology (ENT) and Head-Neck Surgery, Shaarei-Tzedek Medical Center, Jerusalem 9103102, Israel; marugete@gmail.com; 3 Hadasa School of Medicine, Hebrew University, Jerusalem 9112102, Israel; levyron67@gmail.com; 4 The Nursing Administration, Shamir Medical Center, Zeriffin 60930, Israel; yaffak@asaf.health.gov.il

**Keywords:** immigrants, somatization, postpartum, depression, anxiety, mental disorders

## Abstract

We conducted a prospective study, aimed to study whether the prevalence of mental disorders after birth differs by country of origin. Parturient mothers of Ethiopian origin, Former-USSR (FSU) origin, or nonimmigrant, native-Israeli origin (*n* = 974, all Jewish) were recruited in hospitals in Israel and were followed 6–8 weeks and one year after birth. General linear models were used to study the associations between origin and mental health, comparing Ethiopian and FSU origin with native-Israeli. Ethiopian and FSU mothers were more likely to report on somatic symptoms, compared with native-Israeli women. Ethiopian origin was negatively and significantly associated with anxiety in all three interviews (β = −1.281, β = −0.678 and β = −1.072, respectively; *p* < 0.05 in all). FSU origin was negatively associated with depression after birth (β = −0.709, *p* = 0.036), and negatively associated with anxiety after birth and one-year postpartum (β = −0.494, and β = −0.630, respectively). Stressful life events were significantly associated with all mental disorders in the three time points of interviews. Our findings suggest that immigrants tend to express higher mental distress with somatic symptoms. Additional tools are needed for mental distress screening among immigrants.

## 1. Introduction

Postpartum depression (PPD) is the most studied mental disorder in women after delivery. It is considered the most common complication women experience after delivery, affecting about 13% of mothers worldwide (range: 10–25% across countries) [1,2,3]. PPD includes depressive symptoms that can appear any time between pregnancy and 12 months postpartum [4]. Over the past decades, PPD has gained increasing recognition as a major public health issue, and recommendations for screening and management have been adopted by health organizations worldwide [5,6]. Anxiety after birth has gained much less attention, although the prevalence of generalized anxiety disorders in the postpartum period has been evaluated to range between 8% [7,8] and 18%, [9], which is comparable to the rates of postpartum depression [9],. Overall, approximately 30% of primipara women have reported symptoms of depression, anxiety, or stress, but only a third of them were identified by screening for depression [10,11].

Rates of prenatal and postpartum depression were found to be especially high among immigrant populations in several countries [12,13,14]. A systematic review and meta-analysis showed that with screening by Edinburgh Postnatal Depression Scale (EPDS), immigrant women are twice as likely to experience depressive symptomatology in the postpartum period as nonimmigrant women (pooled aOR = 2.10 95% CI: 1.62–2.73) [15].The circumstances that lead to migration and the stress involved with immigration, may negatively affect the psychology of the new mother [14]. Immigrants’ mental health is associated with the country of origin but also with the integration policies of the receiving countries, circumstances of migration, characteristics of the health care system in the receiving country, and time since immigration, as well as the socioeconomic status [16,17,18]. A systematic-review covering 17 low and low-middle income countries revealed 19.8% (95% CI: 19.5–20.0%) weighted mean prevalence of mental disorders [19]. A study of African countries found a mean prevalence of 18.3% (95% CI 17.6–19.1%) of depression and 14.0% (95% CI 12.9–15.2%) of anxiety after birth [20]. In the Amhara region of Ethiopia, 19.8% had probable postpartum common mental disorders [21]. Mothers in general, and immigrants in particular, may be reluctant to disclose depressive symptomology to their health care professionals due to the social stigmatization of women [22,23], as well as the fear that a woman’s ability to be an adequate mother will be challenged [24].

One of the major problems in assessing the prevalence of PPD and anxiety across cultural settings relates to differences in definitions and expression of symptoms. Some studies have suggested that patients from non-Western cultures or developing countries are more likely to report somatic symptoms and deny psychological symptoms, especially if they are from Asian, African, and Hispanic cultures [25,26]. Ethiopians were found to share a typical cluster of cultural idioms centered mainly on three bodily areas that have specific ethno-physiological meanings: the head, the heart, and the stomach [27]. A Chilean study has shown statistically significant association (OR = 3.2) between somatic symptoms and depression [28]. Additionally, a community-based study in China found that widespread pain symptoms significantly increased the likelihood of respondents having depression (OR = 3.47) [29]. Research carried out in Israel on immigrants from the Former Soviet Union found a high rate of somatization, where most common physical complaints included heart or chest pain, feelings of weakness in different parts of the body, and nausea [30]. Somatoform disorders are persistent and especially costly and difficult to prevent and manage. It thus sets major medical, social, and economic challenges [31]. 

Our hypothesis was that manifestations of postpartum mental disorders differ between immigrants and native-born in Israel according to different mental scales. Therefore, we aimed to study the different manifestations of mental disorders in parturient women from three origin groups, namely women of Ethiopian origin (ETH), Former Soviet Union origin (FSU) and native Israeli-born (INB) in a prospective study designed in three waves. Our assumption was that immigrant groups would have an increased prevalence of PPD, anxiety and somatic symptoms, and that there may be a different expression of mental morbidity after delivery among immigrants.

## 2. Methods

We conducted a three-step study of mothers. Mothers were recruited after birth in 3 different hospitals in Israel (Hillel Yaffe, Shamir, and Kaplan Medical Centers) between April and December 2017 and were followed for one year. The study included women older than 18 years who were capable of understanding and completing questionnaires (in Hebrew, Russian, or Amharic) in the postpartum period. The study population included 964 mothers: 484 Israeli-Native-born mothers, 264 Former-USSR origin mothers (212 first generation and 52 second generation immigrants) and 216 Ethiopian origin mothers (191 first generation and 25 second generation immigrants). After delivery, participating women answered an anonymized self-administered questionnaire that included questions about socioeconomic conditions, age, smoking habits, alcohol consumption, living with a spouse and type of delivery. Six to eight weeks after delivery and again after twelve months, the women were re-interviewed by phone. Anxiety and depression were assessed by completing the General Anxiety Disorder 7 Item Scale (GAD-7) and the Edinburgh Postnatal Depression Scale (EPDS), respectively. Seven questions from the SRQ-F questionnaire (the Revised Self-Reporting Questionnaire for Ethiopian origin population) were selected based on experts’ opinion, who built the original SRQ questionnaire and assisted us in selecting the most relevant questions for our research. The questionnaires were used in all 3 stages of the study (SRQ-7).

Between the first and second interview, 433 (44.9%) participants were lost to follow-up, and additional 118 (12.2%) participants were lost to follow-up between the second and third interview. Loss to follow-up was associated with low income (27.5% vs. 22.7%, lost to follow-up vs. participants, respectively, *p* = 0.007) and low education (≤12 years of education 48% vs. 38%, lost to follow-up vs. participants, respectively, *p* = 0.002). No significant differences were found in EPDS, GAD-7 and SRQ-7 scores, between those lost to follow-up and participants in the second interview (not shown). 

### 2.1. Tools for Mental States Assessments

Commonly used tools for screening for postpartum depression and anxiety were the Edinburgh Postpartum Depression Scale (EPDS) [32,33] and Generalized Anxiety Disorder 7-Item Scale (GAD-7) [32], respectively. However, it is not clear how well they identify postpartum depressive symptoms (PPD) and postpartum anxiety in some diverse populations [33,34,35]. The Edinburgh Postpartum Depression Scale (EPDS) is the most commonly used tool for screening depression, which has been validated in various countries, languages, and settings [36,37,38,39]. Cross-cultural variation is demonstrated by different EPDS cut-offs ranging from 7 [40,41] to 12 [42,43]. The questionnaire includes 10 questions on a scale of 0–3. Scoring of 10 or above on the EPDS questionnaire was classified as prevalent depression. 

GAD-7 is a screening instrument which indicates the presence of symptoms of anxiety referred to in the DSM-IV [44]. The questionnaire includes 7 questions on a scale of 0–3, with different cut-offs ranging from 7 [45] to 10 [34,46]. In our study, scoring of 8 or above was classified as prevalent anxiety. The GAD-7 has been translated into several languages and validated in multiple studies [34,47,48] and in Israel [49]. However, a study of Parkerson found that Black/African American participants with high anxiety symptoms scored lower on the GAD-7 than other participants with similar anxiety symptoms [37].

Some studies reported about challenges in screening of PPD and anxiety in specific populations since the different dialects within countries also raises the possibility of idiosyncratic elements, and individual ways of interpreting maternal depression. Several studies found psychological symptoms manifest differently from culture to culture [35,50,51]. This fact indicates the need for the development of assessment instruments for postpartum depression that are validated within various cultures.

The Self-Reporting Questionnaire (SRQ), developed by WHO experts, was designed for screening psychiatric illness, especially in developing countries, regardless of cultural context [52]. This questionnaire has been validated and adjusted among Ethiopians in Ethiopia and Israel, with specific reference to somatic symptoms that are considered as ethno-physiological. We have not found in the literature the use of the SRQ questionnaire in FSU and Israeli-born populations. However, there is evidence in the literature on the use of somatization among these populations to diagnose depression [30,53,54]. The validity of the revised instrument (SRQ-F) was found superior to that of the original instrument (SRQ)26. This questionnaire contains 29 sections and due to its length, 7 questions were selected and included in our study questionnaire (questions in the WHO-SRQ Questionnaire as well as in the SRQ-F: headaches, appetite decrease, unpleasant feelings in the stomach; questions in the SRQ-F questionnaire only: feeling someone tried to harm them, hearing voices, feeling cursed and increase in heart rate), each was scored 0 or 1, and were all summed up (Cronbach’s Alpha: 0.46).

In this study, we used the GAD-7 EPDS and SRQ-7 questionnaires, for identifying anxiety, depression, and somatic disorders among mothers.

### 2.2. Covariates

Origin was defined by the women’s or their mothers’ country of birth (Israeli-Native-Born (INB), Ethiopian Jew (ETH), and Former-USSR Jew (FSU)). Information on a broad range of variables was collected at study intake, 6–8 weeks postpartum and at 12 months postpartum (concurrent variables). Socio-demographic, family history and pregnancy variables measured at study intake included maternal age (grouped into 17–24, 25–29, 30–34, 35–39, and ≥40), religiosity (defined as secular, traditional, orthodox and ultra-orthodox), marital status (married or living with a spouse vs. single, divorced or widowed), maternal years of education (<13 vs. ≥13), income (above, same and lower than the average, a net NIS 12,000 per household), smoking during pregnancy (yes vs. no), obstetric history including conception (spontaneous vs. fertility treatment), birth type (cesarean vs. vaginal), parity (grouped into 1, 2–4 and ≥5), gestational age (grouped into <36, 37–40 and ≥41 weeks), birth weight (grouped into 1501–2500, 2501–4000 and ≥4001 grams), gender, multigestation (yes vs. no), breastfeeding at 6–8 weeks (yes vs. no), and stressful life events in the previous year (assessed by six questions obtained from life events and coping scales during the gestational period (the death of someone in the family, severe diseases, unemployment, disengagement with partner, legal or financial problems or other stressful event; grouped into any vs. none) [55].

### 2.3. Data Analysis

Descriptive characteristics of the study population were presented and compared by origin groups using one-way ANOVA and chi-square tests, as appropriate. Medians of EPDS and GAD-7 scores were presented, and distributions were compared by SRQ-7 as a dichotomous score (0 vs. ≥1), using Mann–Whitney test. The associations between origin and the study scales of mental disorders as continuous variables were assessed using generalized linear models where univariate models were followed by multivariable models, controlling for variables that were significantly associated with the outcomes in univariate models (maternal age, family status, and education). 

Statistical analysis was performed with IBM SPSS Statistics for Windows, Version 25.0 (Armonk, NY, USA: IBM Corp). We present regression coefficients β and *p*-values.

The study protocol was approved by the Institutional Review Board of the Israeli Ministry of Health, IRB #34, 2016, Hillel-Yaffe Medical Center, IRB #102, 2016, Shamir Medical Center, IRB #267, 2016, and Kaplan Medical Center, IRB #77, 2017. 

## 3. Results

Table 1 presents the characteristics of the study participants by three groups of origin. Mothers of the three origin groups were of similar age (31 years). The rate of single motherhood was significantly higher in the ETH group (12.4%) compared to INB and FSU women with 3.5% and 4.2%, respectively (*p* < 0.001). The largest gap between the groups was observed in maternal education; only 33% of ETH had >12 years of education, whereas more than 62% of both other groups had more than 12 years of education. Similarly, ETH women were more likely to come from a household with lower than the average income (47.3% vs. 18.5% and 19.9% among ETH vs. ISR and FSU, respectively). Among married women, low spousal support was reported by 9.5% of ETH women, compared with 4.5% among FSU women and 3.6% among INB women. ETH women had less prenatal care during pregnancy, with 12.7% having none or partial perinatal care, compared with 9.9% among INB and 5.0% among FSU, and higher prevalence of late (>40 week) deliveries (25.9% in ETH group, 13.2% in INB and 15.0% in FSU). Additionally, ETH women had the highest rates of cesarean and instrumental births (35.4%) compared to INB (29.4%) and FSU women (29.9%).

**Figure 1:**
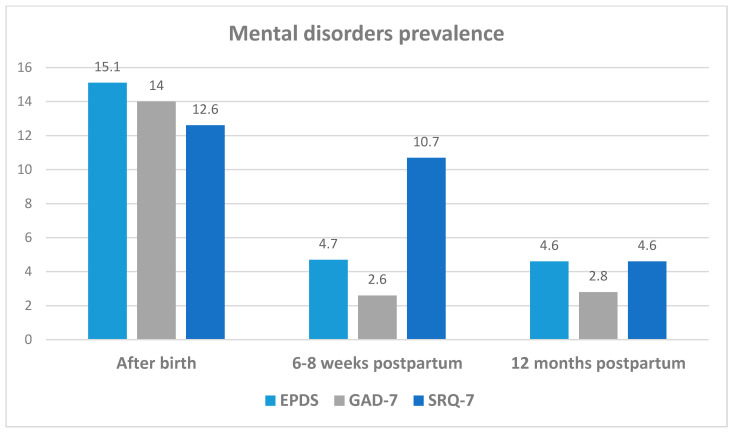
Prevalence of EPDS, GAD-7 and SRQ-7 positive scores by 3 points of time of the study.

Taken together across the study population, as shown in Figure 1, prevalence rates of the three mental conditions studied were the highest right after birth. Positive EPDS and GAD-7 tests were lower and stable in the 6–8 weeks and 1 yr postpartum interviews (4.7% and 2.6–2.8%, respectively), whereas the prevalence of somatic symptoms decreased from 10.7% 6–8 weeks postpartum to 4.6% at 12 months. 

Prevalence rates of positive scores of all three mental disorders by origin in the three time points of the study are presented in Table 2. SRQ scores were higher for the two immigrant groups at all stages of the study. However, the EPDS score was highest among INB in the first phase, but not in the next two phases.

**Figure 2:**
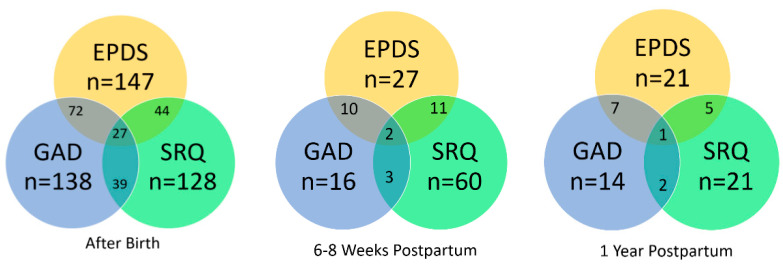
Venn diagrams showing overlap of mental disorders among mothers at each stage (The numbers in the center of each circle represent the total numbers) [56,57].

The overlap of prevalent cases in positive EPDS, GAD-7 and SRQ-7 at each interview is presented in Figure 2. It can be seen at all stages, that over 25% of women who have experienced depressive symptoms (EPDS) have also experienced psychosomatic symptoms (SRQ-7).

Studying the associations between origin and EPDS, GAD-7, and SRQ-7 scores (Table 3), Ethiopian origin was negatively associated with GAD-7 in all three stages (β = −1.281, *p* = 0.001; β = −0.678, *p* = 0.020; and β = −1.072, *p* = 0.007; right after birth, 6–8 weeks, and 12 months postpartum, respectively). FSU origin was negatively associated with EPDS after birth (β = −0.709, *p* = 0.036), and negatively associated with GAD-7, 12 months postpartum (β = −0.630, *p* = 0.056). In contrast, Ethiopian and FSU origin were positively associated with SRQ-7 scores after birth (β = 0.221, *p* = 0.004 and β = 0.121, *p* = 0.066, respectively). Other factors that were associated with SRQ-7 included maternal age of 18–24 years, compared with 35+ years for high score of SRQ-7 after birth (β = 0.244, *p* = 0.019). Low education (<12 years) was associated with SRQ-7 scores in all three stages of the study (β = 0.437, *p* = 0.002; β = 0.450, *p* = 0.030; and β = 0.522, *p* = 0.024; compared with >13 years, respectively). Stressful life events were consistently strongly associated with all mental states at all three stages of the study (EPDS: β = 0.897, *p* < 0.001; β = 0.805, *p* < 0.001; and β = 0.674, *p* < 0.001; GAD-7: β = 0.730, *p* < 0.001; β = 0.357, *p* = 0.002; and β = 0.645, *p* < 0.001; at 0, 6–8 weeks and 1-year postpartum, respectively). The associations with SRQ-7 tended to be somewhat weaker (SRQ-7: β = 0.190, *p* < 0.001; β = 0.192, *p* < 0.001; and β = 0.186, *p* < 0.001, respectively). When a variable of sense of racism was entered into the models, it was found to be a significant risk factor for SRQ-7, but its presence weakened the association between origin and SRQ-7 (not shown).

## 4. Discussion

The present study aimed to investigate the association between migration and mental disorders postpartum. The main finding of this study is that compared to nonimmigrant mothers, mothers of both immigrant groups were more likely to report on somatic symptoms. In this study, we present the prevalence of somatization that was found to vary across the immigrant groups, depending on maternal age, educational level, and life events, in the different three time points of interview. In our first hypothesis, PPD, anxiety and somatic symptoms would be significantly associated with origin and migration because of the stress that has been associated with migration or due to being from an immigrant family and its consequences [14,15,17]. Moreover, psychosomatic symptoms have been reported to be higher in non-Western cultures [25]. Rates of somatization in immigrants has been reported to range between 12.9% and 67% [58]. Likewise, we assumed that mental disorders would manifest differently between origin groups. Our study examined the prevalence of PPD, anxiety and somatic disorders for the three origin groups. We found that psychosomatic symptoms were associated with both EPDS and GAD-7 scores. INB mothers had the highest rate of depression after birth, and the highest prevalence of anxiety after birth and 12 months postpartum. The prevalence of somatic disorders was the highest among immigrants in general and among mothers of Ethiopian origin particularly. However, the prevalence of the three mental disorders was the highest right after birth and declined thereafter. 

In our study, the overall prevalence of positive EPDS (score ≥ 10) 6–8 weeks postpartum was 4.7%, which is identical to the prevalence found in a study of Mother and Child Health Clinics in Israel from 2014–2015 [59], and similar to the prevalence reported by Israel’s largest HMO [60]. These sources support the validation of our research. This prevalence rate is lower than reported by studies from other countries [1,61], representing perhaps cultural differences in reporting, or perhaps a need for a different cut-off score for the Israeli population.

Our findings of the associations between psychosomatic symptoms, EPDS and GAD-7 scores are consistent with worldwide literature and suggest that among immigrants in Western countries, somatization is a manifestation of other mental disorders [25,26,30]. EPDS and GAD-7 median scores were systematically higher among women with any somatic symptom. We found a reasonable association between the median scores of EPDS and GAD-7 with SRQ-7, which suggest that patients with a higher somatic load tend to report higher depression and anxiety scores. Using these interpretations, we found in our analysis a consistent association between somatization, depression and anxiety, which suggests that the SRQ-7 has good construct validity.

Depression is an emotional disorder whose expression differs between the Western and non-Western world [23]. Reports of culture specific complaints have included complaints of ants creeping in the brain by Nigerians [62], and complaints of exhaustion or that hearts are being squeezed and weighed down by Chinese [62]. It has been suggested that hypochondriasis and somatization are expressions of depression in Africa [23]. Studies from Turkey and Pakistan estimated 23–25% of women with somatic disorders had some form of depressive disorder [63,64]. 

Depression, anxiety and somatoform disorders are the most prevalent disorders in primary care settings [65]. Indeed, in many patients consulting primary care physicians, mental health problems manifest themselves in the form of physical complaints rather than psychological or emotional ones [66]. A Spanish study of patients in primary care units found 28.8% of the patients had somatoform disorder and 11.5% presented comorbidity between affective, anxiety, and somatoform disorders [67]. 

Our findings suggest that somatic symptoms are probably a core component of the depressive syndrome; our results show a strong association of somatization with depression and with anxiety, and that they add another dimension not fully appreciated by the other scales. These results are consistent with other studies, which found that in over 50% of cases, comorbidities existed between depression, anxiety and somatization [25,68]. Studies from Puerto Rico and Taiwan found a significant association between headache and psychiatric evaluation of depression [69,70,71]. Depression also involves conspicuous somatic symptoms of decreased appetite [72], heartbeat [73] and paranoia (including feeling someone wants to harm you) [74]. 

We examined the course of variation in the rate of mental disorders throughout the postpartum year. To avoid selection bias, we examined rates of depression among women who participated in the second phase of the study compared with women lost to follow-up and we found similar depression rates (15.2% vs. 14.7%, respectively). In our study, the rate of mental disorders was the highest after birth, with a subsequent decline in the rate of disorders in the different origin groups. Few studies considered the variation of PPD rates, yet there is no consensus about the course of PPD during the first year postpartum. Although some studies reported remission of PPD in the first year postpartum [75,76], other reported an increase. A national Swedish study found PPD rates of 11.1% two months postpartum and 13.7% one-year postpartum [77]. However, the incidence rate of PPD in late pregnancy or early postpartum among Vietnamese mothers was 13%, and 70% of them recovered within the first postpartum year, without receiving formal mental health care [78].

The present study found that the most persistent variable associated with all postnatal mental disorders was stressful life events in the past year. Our results are consistent with numerous epidemiological studies [79,80,81] which have suggested a multifactorial etiology for postpartum mental disorders including stressful life events, marital status [82,83], low income and education, young maternal age [84,85,86], and experiences of discrimination [87]. Discrimination may contribute to disparities in prevalence of depression and other adverse health outcomes. A review of population-based studies suggests that discrimination is associated with poorer physical and mental health [88] and higher scores of depression [89]. Similar to our study, discrimination was found positively related to depression in studies among African American and White women in the USA [89,90], as well as lack of support from spouse, which was found to be a significant risk factor for maternal depression in different cultures [79,82].

Our study’s limitations include the lack of information on maternal mental state while pregnant which can influence postpartum mental health [82,83] and lack of information on duration of depression and maternal recovery. Other limitations include that the SRQ method measurement has very low reliability, and the lack of validity of the SRQ questionnaire in the Israeli- and USSR-born populations. In addition, we lost 433 (44.9%) women in the second stage. Similar to characteristics of participants lost to follow-up found in the literature [91], mothers lost to follow-up were less educated and with lower incomes, characteristics which are related to depression. However, women lost to follow-up did not have different rates of mental disorders. Furthermore, the degree of acculturation was found to be positively associated with psychiatric disorders, particularly affective disorders. However, we could not estimate the degree of maternal acculturation in Israel. Finally, our research examined the scores of seven questions on psychosomatic symptoms from a broader questionnaire (SRQ-F). Future studies are needed to support the use of this shortened questionnaire.

Our study has several strengths. First, the present study is among the first to examine the validity of psychosomatic symptoms across cultural groups. Furthermore, our findings support a vast literature illuminating cultural-based differences in expression of mental psychopathology [23,92]. Cross-origin differences observed between total EPDS, GAD-7 and SRQ-7 scores highlight the need for culturally-sensitive mental disorders screening and tools.

In conclusion, although psychological distress has been observed to be higher among immigrants than among native populations, little is known about the prevalence, number, and severity of somatic symptoms associated with psychological distress in the immigrant population. The findings presented here, based on a unique database representing Jewish Israeli-, FSU- and Ethiopia-born women in Israel, extend our knowledge in the field. The findings of the study raise the question of whether screening tests used to detect depression at MCHC and in the HMO clinics are adapted to Israeli mothers in general and immigrant mothers, or whether a cultural adjustment is needed to improve and more accurately identify mental disorders among Israeli populations. Somatization is a challenge for health professionals due to its vague nature. In this regard, clinical management of immigrant patients should include further efforts to address emotional distress, with special attention to cultural differences. In addition, more research is needed to explore this potential relationship between psychosomatic symptoms and depression/anxiety further.

## Figures and Tables

**Table 1:** Characteristics of study participants by origin.

	INB * N = 480 % (*n*)	ETH N = 210 % (*n*)	FSU N = 262 % (*n*)	*p* X^2^/F
Total				
Maternal age, years (mean ± SD)	30.9 ± 4.9	30.9 ± 5.2	31.2 ± 4.9	0.693
Religiosity				
Secular	38.8 (186)	16.4 (33)	63.3 (162)	<0.001
Traditional	41.0 (197)	59.7 (120)	28.9 (74)	
Orthodox	14.2 (68)	20.9 (42)	4.7 (12)	
Ultra-Orthodox	6.0 (29)	3.0 (6)	3.1 (8)	
Maternal Status				
Married	96.5 (463)	87.6 (184)	95.8 (251)	<0.001
Unmarried	3.5 (17)	12.4 (26)	4.2 (11)	
Maternal Education group				
1–12	34.5 (164)	67.0 (130)	37.2 (96)	<0.001
>12	65.5 (311)	33.0 (64)	62.8 (162)	
Income				
Above average	46.5 (221)	19.5 (36)	43.0 (108)	<0.001
Same as average	34.9 (166)	33.2 (61)	37.1 (93)	
Lower than average	18.5 (88)	47.3 (87)	19.9 (50)	
Insurance				
Yes	94.0 (426)	73.7 (129)	86.8 (197)	<0.001
No	6.0 (27)	26.3 (46)	13.2 (30)	
Pregnancy				
Planned	73.2 (347)	74.3 (150)	78.7 (203)	0.255
Unplanned	26.8 (127)	25.7 (52)	21.3 (55)	
Pregnancy				
Spontaneous	88.1 (420)	94.4 (185)	88.0 (227)	0.037
Fertility treatment	11.9 (57)	5.6 (11)	12.0 (31)	
Perinatal care during pregnancy				
Full	90.1 (429)	87.3 (178)	95.0 (249)	0.011
No/partial	9.9 (47)	12.7 (26)	5.0 (13)	
Birth type				
Spontaneous	70.6 (333)	64.6 (135)	70.0 (180)	0.202
Instrumental	5.9 (28)	6.7 (14)	3.1 (8)	
Cesarean	23.5 (111)	28.7 (60)	26.9 (69)	
Parity (mean ± SD)	2.20 ± 1.2	2.24 ± 1.4	2.18 ± 1.1	0.874
1	34.3 (155)	35.3 (67)	31.3 (78)	0.559
2–4	61.1 (276)	57.4 (109)	63.1 (157)	
>4	4.6 (21)	7.4 (14)	5.6 (14)	
Gestational age (mean ± SD)	39.0 ± 1.8	39.2 ± 1.8	38.9 ± 1.6	0.061
Gestational age group				
<36	5.9 (22)	6.3 (10)	5.5 (12)	0.007
37–40	80.9 (300)	67.8 (107)	79.5 (175)	
>40	13.2 (49)	25.9 (41)	15.0 (33)	
Birth weight (mean ± SD)	3168 ± 509	3122 ± 509	3272 ± 543	0.004
Birth weight group (gr.)				
<2500	8.3 (40)	11.0 (23)	8.8 (23)	0.525
>2500	91.7 (440)	88.0 (186)	91.2 (238)	
Apgar 1 (mean ± SD)	8.90 ± 0.6	8.87 ± 0.5	8.96 ± 0.2	0.148
Apgar 5 (mean ± SD)	10.05 ± 2.8	9.93 ± 0.2	9.94 ± 0.3	0.679
Gender				
Male	52.0 (246)	50.7 (107)	46.9 (123)	0.418
Female	48.0 (227)	49.3 (104)	53.1 (139)	
Multigestation				
Yes	4.2 (20)	2.8 (6)	2.3 (6)	0.345
No	95.8 (458)	97.2 (206)	97.7 (257)	
Smoking during pregnancy				
Yes	9.3 (42)	14.0 (27)	9.2 (23)	0.155
No	90.7 (412)	86.0 (166)	90.8 (227)	
Alcohol during pregnancy				
Yes	7.9 (36)	5.9 (11)	17.7 (44)	<0.001
No	92.1 (418)	94.1 (175)	82.3 (205)	
Breastfeeding at 6–8 weeks				
Exclusive	39.1 (109)	57.4 (66)	46.1 (70)	0.001
Partial	13.6 (38)	18.3 (21)	15.7 (24)	
None	47.3 (132)	24.3 (28)	38.2 (58)	
Spouse support (among married)				
High	96.4 (426)	90.5 (153)	95.5 (231)	0.015
Low	3.6 (14)	9.5 (16)	4.5 (11)	
Stressful events				
Yes	32.4 (155)	35.4 (68)	39.4 (98)	0.177
No	67.6 (303)	64.6 (124)	60.6 (151)	
Racism (mean ± SD)	11.04 ± 2.4	14.05 ± 7.5	11.81 ± 4.1	<0.001

* ETH = Ethiopian origin, FSU = Former-USSR origin, INB = nonimmigrant, native-Israeli origin.

**Table 2:** Rate of positive dichotomous scores of EPDS, GAD-7, SRQ-7 by origin in 3 time points of the study.

#	INB %	ETH %	FSU %
*After birth*			
EPDS ≥ 10	17.5	14.0	11.8 **
GAD-7 ≥ 8	15.9	11.5 **	12.6 **
SRQ-7 any symptom	9.6	17.7 **	14.0 *
*6–8 weeks postpartum*			
EPDS ≥ 10	3.9	5.1	5.9
GAD-7 ≥ 8	2.5	2.6 *	2.6
SRQ-7 any symptom	8.0	13.9	13.2
*12 months postpartum*			
EPDS ≥ 10	3.2	7.9	4.7
GAD-7 ≥ 8	3.2	2.3 **	2.3 *
SRQ-7 any symptom	3.2	7.9	4.7

# ETH = Ethiopian origin, FSU = Former-USSR origin, INB = nonimmigrant, native-Israeli origin; * *p* < 0.1, ** *p* < 0.05.

**Table 3:** Generalized linear models predicting EPDS, GAD-7 and SRQ-7 in 3 points of time of the study.

	EPDS β	GAD-7 β	SRQ-7 (SUM) β
*After birth*			
INB	Ref.	Ref.	Ref.
ETH	−607	−1.281 **	0.221 **
FSU	−0.709 **	−0.494 **	0.121 *
Age 18–24 (ref. > 35)	1.039 *	0.780	0.244 **
Age 25–34 (ref. > 35)	−0.922 **	−0.461	−0.148 **
Married (ref. single)	1.510 **	−0.996	−0.164
Education < 12 (ref. > 13)	1.076	0.302	0.437 **
Education = 12 (ref. > 13)	−0.261	−0.462	0.028
Trauma events	0.897 ***	0.730 ***	0.190 ***
*6–8 weeks postpartum*			
INB ^#^	Ref.	Ref.	Ref.
ETH ^#^	0.131	−0.678 **	0.038
FSU ^#^	0.129	−0.099	0.106
Age 18–24 (ref. > 35)	0.509	−0.223	0.255
Age 25–34 (ref. > 35)	0.192	0.256	0.149
Married (ref. single)	−0.044	0.796	0.060
Education < 12 (ref. > 13)	0.306	0.336	0.450 **
Education = 12 (ref. > 13)	−0.258	0.027	0.030
Trauma events	0.805 ***	0.357 **	0.192 ***
*12 mo. Postpartum*			
INB ^#^	Ref.	Ref.	Ref.
ETH ^##^	−0.112	−1.072 **	0.064
FSU ^#^	0.029	−0.630 *	0.101
Age 18–24 (ref. > 35)	−0.397	−0.284	0.263
Age 25–34 (ref. > 35)	−0.532	−0.330	0.069
Married (ref. single)	−0.044	0.495	0.044
Education < 12 (ref. > 13)	0.124	0.592	0.522 **
Education = 12 (ref. > 13)	0.872 **	0.330	0.274 **
Traumatic life events	0.674 ***	0.645 ***	0.186 ***

* *p* < 0.1, ** *p* < 0.05, ***p < 0.001; ^#^ FSU = Former-USSR origin, INB = nonimmigrant, ^##^ ETH = Ethiopian origin, native-Israeli origin.

## Data Availability

No appliable.
